# Telocytes in liver regeneration: possible roles

**DOI:** 10.1111/jcmm.12355

**Published:** 2014-07-31

**Authors:** Fei Wang, Yang Song, Yihua Bei, Yingying Zhao, Junjie Xiao, Changqing Yang

**Affiliations:** a Division of Gastroenterology and Hepatology, Digestive Disease Institute, Tongji Hospital, Tongji University School of MedicineShanghai, China; b Regeneration Lab and Experimental Center of Life Sciences, School of Life Science, Shanghai UniversityShanghai, China; c Innovative Drug Research Center of Shanghai UniversityShanghai, China

**Keywords:** telocytes, liver regeneration, hepatocytes, CK-19 positive-hepatic stem cells, CD34, PDGFR-α, ß, EdU, PCNA

## Abstract

Telocytes (TCs) are a novel type of interstitial cells which are potentially involved in tissue regeneration and repair (http://www.telocytes.com). Previously, we documented the presence of TCs in liver. However, the possible roles of TCs in liver regeneration remain unknown. In this study, a murine model of partial hepatectomy (PH) was used to induce liver regeneration. The number of TCs detected by double labelling immunofluorescence methods (CD34/PDGFR-α, CD34/PDGFR-ß and CD34/Vimentin) was significantly increased when a high level of hepatic cell proliferation rate (almost doubled) as shown by 5-ethynyl-2′-deoxyuridine (EdU) immunostaining and Western Blot of Proliferating cell nuclear antigen (PCNA) was found at 48 and 72 hrs post-PH. Meanwhile, the number of CK-19 positive-hepatic stem cells peaked at 72 hrs post-PH, co-ordinating with the same time-point, when the number of TCs was most significantly increased. Taken together, the results indicate a close relationship between TCs and the cells essentially involved in liver regeneration: hepatocytes and stem cells. It remains to be determined how TCs affect hepatocytes proliferation and/or hepatic stem cell differentiation in liver regeneration. Besides intercellular junctions, we may speculate a paracrine effect *via* ectovesicles.

## Introduction

Liver possesses a remarkable capacity to regenerate after toxic injury, virus infection, ischaemia and surgical resection [1]. Liver regeneration is typically mediated by the proliferation of remaining cells, with hepatocytes as the first to enter cell cycle and reach the peak of DNA synthesis, while non-parenchymal cells (Kupffer cells, biliary epithelial cells, hepatic stellate cells and endothelial cells, *etc*.) are later to proliferate [2]. Unfortunately, the proliferative capacity of hepatocytes is usually impaired upon severe or chronic liver injuries. In such circumstances, liver stem/progenitor cells are assumed to be activated and contribute to liver regeneration by differentiating to mature cells [3]. Considering that different types of hepatic cells participate in liver regeneration in an orchestrate manner, understanding how these cells work together as well as the regulatory mechanisms for their biological processes will help advance the basic knowledge on molecular and cellular aspects of liver regeneration.

Our group has previously documented the presence in mice liver of a distinct type of interstitial cell termed telocyte (TC) [4]. TCs were firstly identified by Popescu's group [5–11], and then have been documented in the interstitial space of various organs and tissues in mammals [12–20]. TCs are characterized by a small cell body and extremely long prolongations named telopodes (Tps) with an alteration of thin segments (podomers) and dilated segments (podoms) [21–26]. Besides the established microRNA signatures [27] and gene profiles [28], the proteomic features [29] as well as chromosome 1 gene profile [30] of TCs have been identified. Increasing evidence indicated the potential roles of TCs in tissue regeneration and repair (heart, muscle and skin) by forming a complex network with neighbour cells and releasing shed vesicles and exosomes, thus regulating intercellular signalling that might be essentially involved in regeneration/repair [7,8,16,17,25,31–34]. However, it remains to be determined the possible roles of TCs in the control of liver regeneration.

Thus, the aims of the present study were to investigate the roles of TCs in liver regeneration by using a murine model of partial hepatectomy (PH).

## Materials and methods

### Animals

Eight-week-old specific pathogen-free male C57BL/6 mice were purchased from SLAC Laboratory Animal Center, Shanghai (Shanghai, China). Mice were maintained in a temperature-controlled room on a 12 hrs light/dark cycle, with free access to water and standard chow. Mice were anaesthetized by 1% pentobarbital sodium intraperitoneal injection (50 mg/kg). Seventy Percent PH was performed on anaesthetized mice by removing the median and left lobes of the liver. At various time-points post-PH (0, 12, 24, 48, 72, 96, 120 and 168 hrs), totally 40 mice were killed (five mice for every group). The mice of 0 hr (quiescent liver) group were used as baseline. Residual liver lobes and body weight were weighed at the time of killing, and the ratio of residual liver lobes weight to body weight was calculated to evaluate the regeneration of liver mass. Frozen liver sections were used for immunofluorescent staining and EdU (5-ethynyl-2′-deoxyuridine) immunostaining. The rest of liver tissues were collected and stored at −80°C for Western blot analysis. This study was approved by the local ethical committees and all animal experiments were conducted under the established guidelines on the use and care of laboratory animals for biomedical research published by National Institutes of Health (No. 85-23, revised 1996).

### EdU immunostaining

Mice were injected intraperitoneally with 50 mg/kg of EdU (C10314-3; Riobio, Guangzhou, China) 1 hr before killed. After washed with PBS for 15 min., frozen sections were fixed in paraformaldehyde for 30 min. After fixed, frozen sections were washed with PBS for three times, and then incubated with Cell-Light™ EdU Apollo®488 In Vivo Imaging Kit (C10314-3; Riobio). After incubation with EdU kit, sections were washed with PBS for three times, then incubated with DAPI (4′,6-diamidino-2-phenylindole, 1:500 dilution; Life Technology, Carlsbad, CA, USA). The images were taken under an amplification of 400× with confocal laser scanning microscope (LSM 710; Carl Zeiss MicroImaging GmbH, Jena, Germany). The results were expressed as EdU-positive cell number per mm^2^.

### Western blot analysis

Liver tissues were lysed with RIPA lysis buffer (Beyotime Institute of Biotechnology, Haimen, China). Equal amounts of 30 μg of total protein were subjected to electrophoreses on 10% SDS-PAGE gels, transferred to PVDF membranes and incubated with anti-PCNA (Proliferating cell nuclear antigen, 10205-2-AP, 1:1000; Proteintech, Chicago, IL, USA) or anti-β-actin (M1210-2, 1:1000; HuaAn, Hangzhou, China) primary antibodies. After incubated with the corresponding HRP-conjugated secondary antibodies, protein bands were visualized using enhanced chemiluminescence system (Pierce Biotechnology Inc., Rockford, IL, USA) with ChemiDoc XRS Plus luminescent image analyzer (Bio-Rad, Hercules, CA, USA). Densitometric analysis of protein bands was performed with Image Lab software (Bio-Rad). Loading volume of each sample was normalized by β-actin protein band density.

### Immunofluorescent staining

For detection of TCs in liver, double immunofluorescent staining for CD34/PDGFR-α or CD34/PDGFR-β or CD34/Vimentin was used. Briefly, frozen sections (6 μm thick) were mounted on Superfrost Plus slides (Shitai, China), and then fixed in paraformaldehyde for 15 min. After washed with PBS for three times, sections were pre-incubated in PBS supplemented with 10% goat serum for 1 hr, and then incubated overnight at 4°C with rabbit polyclonal anti-PDGF Receptor-α (ab61219; Abcam, Cambridge, UK) and rat monoclonal anti-CD34 (ab8158; Abcam) primary antibodies. Both antibodies were diluted by 1:100 in 1× PBS with 0.25% Triton X-100. After that, sections were exposed for 1 hr to goat anti-rat labelled with FITC (sc-2011; Santa Cruz, Dallas, TX, USA) and goat anti-rabbit labelled with rhodamine (sc-362262; Santa Cruz) secondary antibodies diluted by 1:200 in the same buffer. Finally, sections were stained with DAPI (ProLong® Gold; Life Technology). The same protocol was used in Rabbit monoclonal to PDGF Receptor-β (ab32570; Abcam) and rat monoclonal anti-CD34 (ab8158; Abcam) double labelling staining, and Rabbit monoclonal to Vimentin (ab92547, 1:100; Abcam) and rat monoclonal anti-CD34 (ab8158; Abcam) double labelling staining. The images were taken under an amplification of 400× with confocal laser scanning microscope (LSM 710; Carl Zeiss MicroImaging GmbH). For detection of hepatic stem cells, immunofluorescent staining for CK-19 was conducted according to the same protocol as described above except that rabbit monoclonal anti-CK-19 primary antibody (ab52625, 1:50; Abcam) was used instead.

### Statistical analysis

All analyses were evaluated using SPSS 19.0. Data are expressed as mean ± SEM. For parametric data, statistical significance was determined with one-way anova test followed by two-tailed Student's *t*-test. For non-parametric data, statistical significance was determined with Mann–Whitney *U*-test. 95% confidence intervals were presented. *P* < 0.05 was considered statistically significant.

## Results

**Fig. 1:**
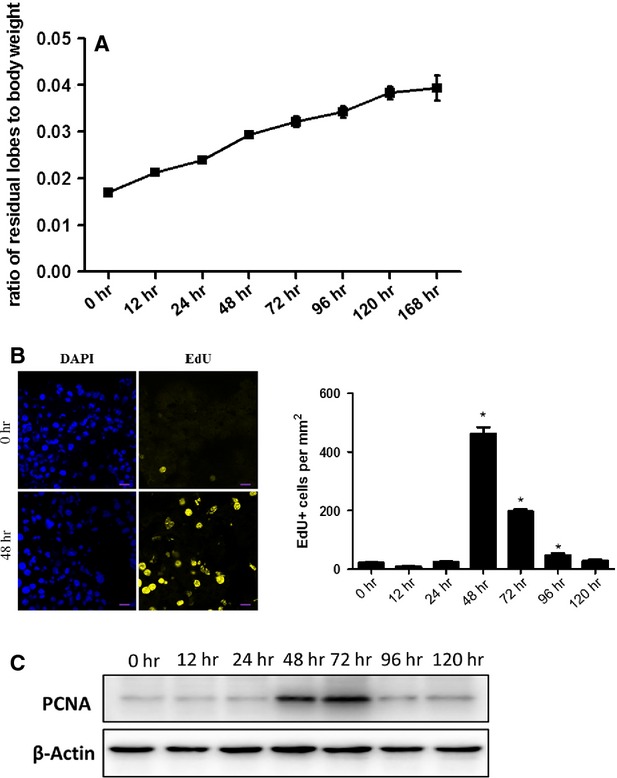
Liver regeneration post-PH. (**A**) The ratio of residual liver lobes weight to body weight post-PH. (**B**) EdU (yellow) immunostaining was performed to evaluate the proliferative cells in liver post-PH. Representative images of EdU-positive cells at 48 hrs post-PH were shown on the left. Quantitative analysis of EdU-positive cells/mm^2^ at various time-points post-PH was shown on the right. Original magnification 400×; scale bar = 20 μm. (**C**) Western blot analysis for PCNA in liver post-PH. *P < 0.05.

As shown in Figure 1A, the ratio of residual liver lobes weight to body weight was gradually elevated within 168 hrs post-PH. EdU immunostaining was performed to further investigate the proliferative effect of liver regeneration post-PH. As shown in Figure 1B, the number of EdU-positive cells/mm^2^ was significantly increased at 48 hrs [*P* = 7.09 × 10^−11^, 95% CI = (418.24, 463.89)] and 72 hrs [*P* = 1.49 × 10^−11^, 95% CI = (168.47, 183.45)] post-PH, accompanied by a remarkable increase of PCNA protein level in liver (Fig. 1C), indicating a high level of cell proliferation rate at 48 and 72 hrs post-PH.

**Fig. 2:**
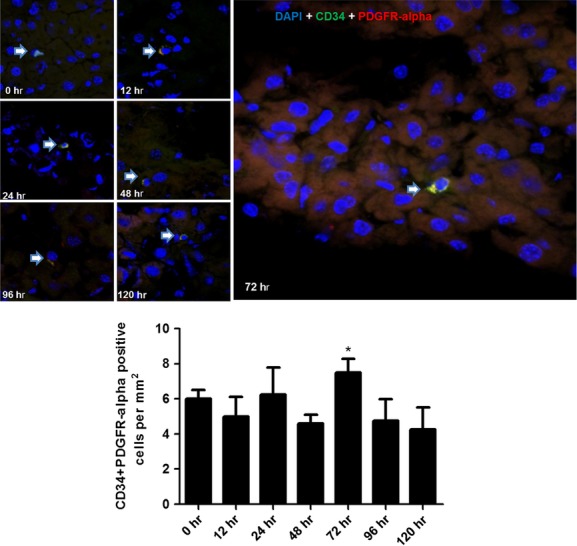
Detection for TCs by double labelling immunofluorescence methods (CD34/PDGFR-α). Detection for TCs by CD34/PDGFR-α double immunofluorescence labelling in liver post-PH. Confocal laser scanning microscopy: double labelling immunofluorescence shows CD34 (green) and PDGFR-α (red) double-positive cells (pointed with arrows). Nuclei were counterstained with DAPI (blue). Original magnification 400×; scale bar = 20 μm. *P < 0.05.

**Fig. 3:**
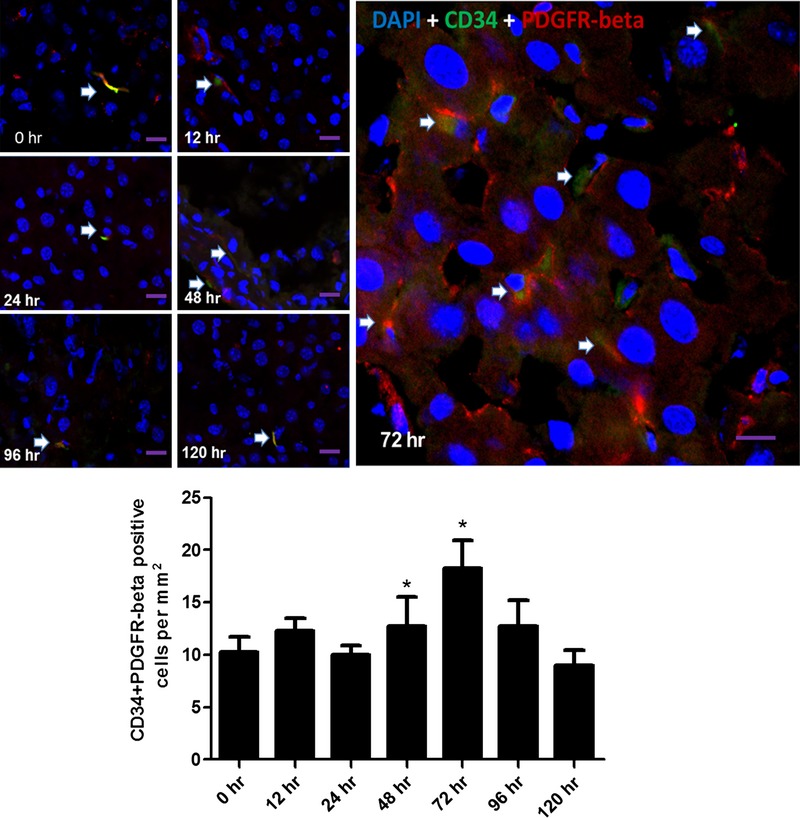
Detection for TCs by double labelling immunofluorescence methods (CD34/PDGFR-ß). Detection for TCs by CD34/PDGFR-ß double immunofluorescence labelling in liver post-PH. Confocal laser scanning microscopy: double labelling immunofluorescence shows CD34 (green) and PDGFR-ß (red) double-positive cells (pointed with arrows). Nuclei were counterstained with DAPI (blue). Original magnification 400×; scale bar = 20 μm. *P < 0.05.

**Fig. 4:**
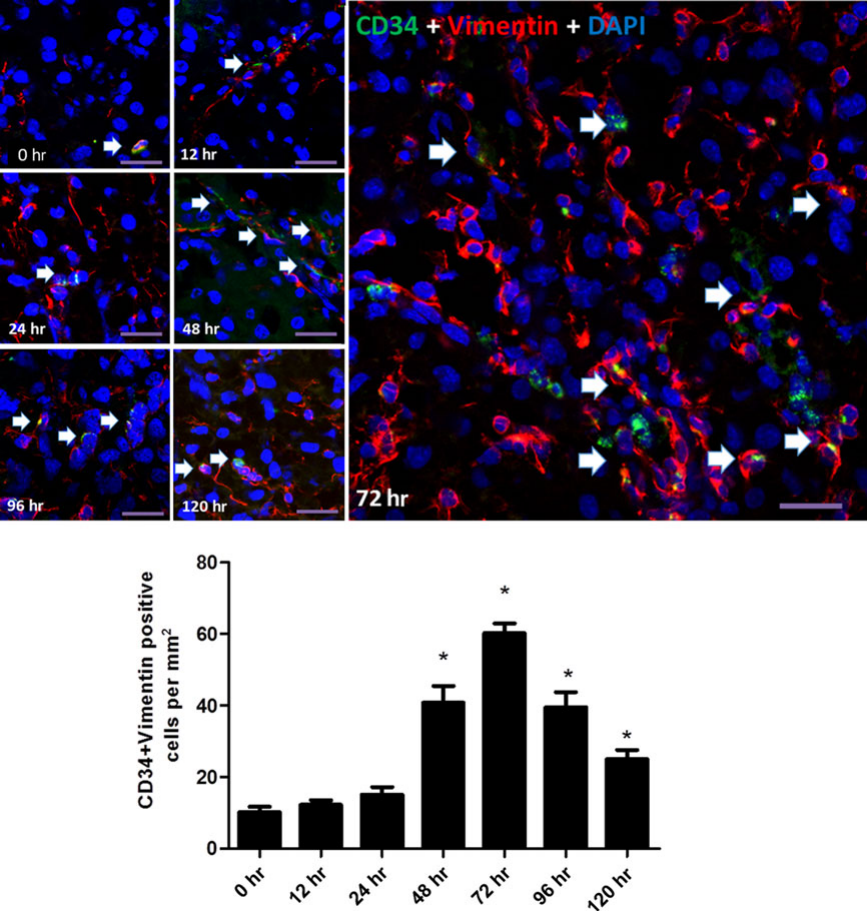
Detection for TCs by double labelling immunofluorescence methods (CD34/Vimentin). Detection for TCs by CD34/Vimentin double immunofluorescence labelling in liver post-PH. Confocal laser scanning microscopy: double labelling immunofluorescence shows CD34 (green) and Vimentin (red) double-positive cells (pointed with arrows). Nuclei were counterstained with DAPI (blue). Original magnification 400×; scale bar = 20 μm. *P < 0.05.

To detect TCs in mice liver, three different double labelling immunofluorescence methods (CD34/PDGFR-α, CD34/PDGFR-ß and CD34/Vimentin) were conducted. The number of CD34/PDGFR-α double-positive cells was significantly increased at 72 hrs [*P* = 0.012, 95% CI = (0.42, 2.57)] post-PH (Fig. 2), and significant increased number of CD34/PDGFR-ß double-positive cells was observed at 48 hrs [*P* = 0.006, 95% CI = (1.49, 6.12)] and 72 hrs [*P* = 0.001, 95% CI = (4.46, 11.53)] post-PH (Fig. 3), while the increase in CD34/Vimentin double-positive cells was observed at 48 hrs [*P* = 2.36 × 10^−16^, 95% CI = (25.38, 35.63)], 72 hrs [*P* = 1.36 × 10^−22^, 95% CI = (45.16, 54.84)], 96 hrs [*P* = 1.53 × 10^−16^, 95% CI = (24.41, 34.09)] and 120 hrs [*P* = 1.87 × 10^−9^, 95% CI = (9.91, 19.59); Fig. 4], corresponding to the proliferative peak time-point of liver regeneration post-PH.

**Fig. 5:**
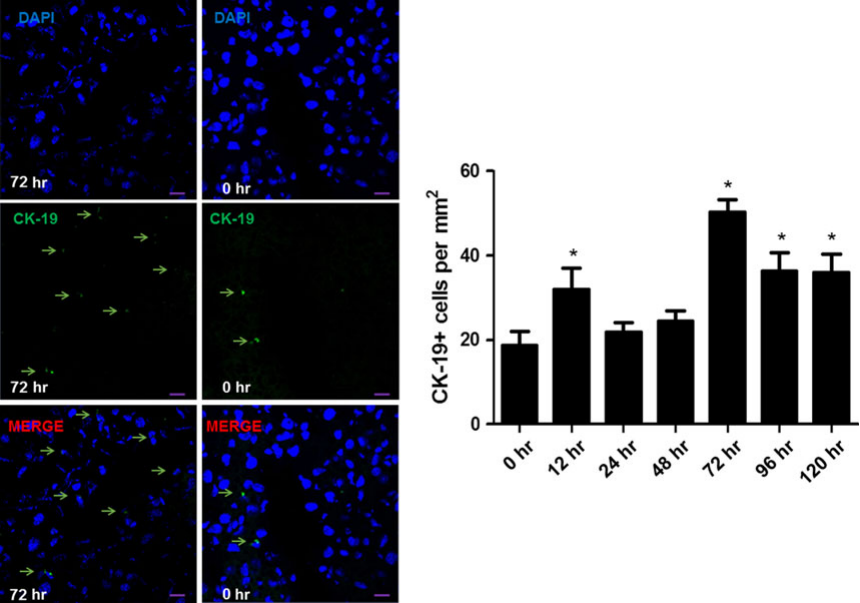
Detection for hepatic stem cells by immunofluorescent staining for CK-19. Representative images of CK-19 (green) positive cells in liver (pointed with arrows). Nuclei were counterstained with DAPI (blue). Original magnification 400×; scale bar = 20 μm. *P < 0.05.

To investigate the quantitative change in hepatic stem cells post-PH, immunofluorescent staining for CK-19 was performed. As shown in Figure 5, the number of CK-19-positive cells was most significantly increased at 72 hrs [5.82 × 10^−7^, 95% CI = (26.39, 36.60)] post-PH, at which the most remarkable increase in TCs number in liver post-PH also appeared.

## Discussion

Our study shows that the number of TCs detected by double labelling immunofluorescence methods (CD34/PDGFR-α, CD34/PDGFR-ß and CD34/Vimentin) was significantly increased at the proliferative peak period of liver regeneration. Meanwhile, the number of CK-19 positive-hepatic stem cells peaked at 72 hrs post-PH, co-ordinating with the time-point when the number of TCs was most significantly increased. These results indicate a close relationship between TCs and hepatocytes and/or stem cells essentially involved in liver regeneration.

Liver regeneration is a highly orchestrate response to the loss of effective liver mass after liver resection or toxic injury [1]. The proliferation of remaining hepatocytes and the differentiation of liver stem cells are considered two main mechanisms for new hepatocyte production [2,3]. CK-19 is a typical marker for adult hepatic stem cells that are activated during liver injuries [3]. Our present study demonstrated a peak of cell proliferation 48–72 hrs post-PH in mice, accompanied by an elevated number of TCs in liver. This suggests that TCs may be probably involved in the proliferative capacity of hepatocytes during liver regeneration. TCs were found to be connected with other cells including stem cells, for example in heart [5], lungs [24], liver [4], skin [8], eye [11], *etc*. However, the roles of TC in the proliferative capacity of hepatocytes remain to be further clarified in liver regeneration.

In the present study, the increased number of TCs 72 hrs after PH was accompanied by a corresponding increase in hepatic stem cells. Previous studies documented the presence of TCs in heart, lung, liver, skeletal muscle, skin and eye in tandem with stem cells [4,5,8,11,24,25]. TCs are responsible for releasing shed vesicles and exosomes, regulating the transcriptional activity and activation of stem cells, thus contributing to stem cell-mediated tissue regeneration/repair [7,8,17,24,25,35]. Under certain severe or chronic injury conditions, the differentiation of hepatic stem cells to mature cells represents another mechanism of liver regeneration [3], whereas it remains to be determined how TCs exert the functional effects on hepatic stem cell-mediated liver regeneration.

Several limitations of the present study should be highlighted. TCs, as a new type of interstitial cells, are characterized by extremely long and thin prolongations called Tps extending from the cell body [5]. Although hepatic TCs are reside in Disse's space like hepatic stellate cells, TCs have very long Tps as indicated in our previous work [4] and also have specific biomarkers (double-positive for CD34 and vimentin, or PDGF-α, or PDGF–β), making them different from hepatic stellate cells [4]. We noticed that several reports indicated CD34+ expressed markers for stellate cells in human foetal liver (not adult) [36], thus it is highly desirable to isolate hepatic TCs and compare their immunofluorescent features to hepatic stellate cells directly. Moreover, similar to reports in other organs like heart and lung [27–29], the comparisons of microRNA signature, gene profile and proteome between hepatic TCs and hepatic stellate cells are needed. In addition, liver regeneration following PH is mainly achieved by hepatocyte replication although a small yet significant propotation of newly formatted hepatocytes may generated from hepatic stem cells [37]. Interestingly, based on the results from our study, the peak activity of cell replication was at 48 hrs, whereas the peak in TCs and stem cell activity was at 72 hrs (although there was considerable overlap), making it possible that TCs might have a more important role in regeneration mediated by hepatic stem cells compared to that by hepatocytes. Thus, it would be interesting to inhibit hepatocyte proliferation using 2-acetylaminofluorene before PH (2AAF/PH) to identify the role of TCs in liver regeneration mediated mainly by the activation of hepatic stem cells [37]. Moreover, a direct evidence of relationship between TCs and CK-19 positive cells is also required in the future.

In conclusion, our study indicates a potential role of TCs in liver regeneration, probably through their close relationship with hepatocytes and hepatic stem cells or a paracrine effect *via* ectovesicles. Three possibilities of the relationship between TCs and the proliferating cells in PH could be proposed: (*i*) the co-ordinating increase in TCs and proliferating cells in PH is just a coincidence; (*ii*) the increased TCs is just associated with the increase in proliferating cells in PH and (*iii*) the increased TCs is the cause for the increase in proliferating cells in PH. Considering that CD34/PDGFR-α, CD34/PDGFR-ß and CD34/Vimentin positive TCs after PH increase with an accompanying increase in hepatocytes and hepatic stem cells, we speculate that TCs participate in liver regeneration either *via* intercellular junctions or a paracrine effect *via* ectovesicles though the direct evidence of relationship between TCs and hepatocytes/hepatic stem cells is required in the further studies. Further understanding the molecular and cellular mechanisms by which TCs affect hepatocytes proliferation and/or hepatic stem cells differentiation in liver regeneration will help establish novel therapeutic strategies for liver failure.
